# Towards feedback-controlled nanomedicines for smart, adaptive delivery

**DOI:** 10.1177/1535370218800456

**Published:** 2018-09-11

**Authors:** Stephen J. Jones, Annette F. Taylor, Paul A Beales

**Affiliations:** 1 School of Chemistry and Astbury Centre for Structural Molecular Biology, University of Leeds, Leeds LS2 9JT, UK; 2 Department of Chemical and Biological Engineering, University of Sheffield, Sheffield S1 3JD, UK

**Keywords:** Drug delivery, chronobiology, controlled release, nanoreactors, bottom-up synthetic biology, pharmacokinetics

## Abstract

Nanomedicines for controlled drug release provide temporal and spatial regulation of drug bioavailability in the body. The timing of drug release is usually engineered either for slow gradual release over an extended period of time or for rapid release triggered by a specific change in its physicochemical environment. However, between these two extremes, there is the desirable possibility of adaptive nanomedicines that dynamically modulate drug release in tune with its changing environment. Adaptation and response through communication with its environment is a fundamental trait of living systems; therefore, the design of biomimetic nanomedicines through the approaches of bottom-up synthetic biology provides a viable route to this goal. This could enable drug delivery systems to optimize release in synchronicity with the body’s natural biological rhythms and the personalized physiological characteristics of the patient, e.g. their metabolic rate. Living systems achieve this responsiveness through feedback-controlled biochemical processes that regulate their functional outputs. Towards this goal of adaptive drug delivery systems, we review the general benefits of nanomedicine formulations, provide existing examples of experimental nanomedicines that encapsulate the metabolic function of enzymes, and give relevant examples of feedback-controlled chemical systems. These are the underpinning concepts that hold promise to be combined to form novel adaptive release systems. Furthermore, we motivate the advantages of adaptive release through chronobiological examples. By providing a brief review of these topics and an assessment of the state of the art, we aim to provide a useful resource to accelerate developments in this field.

**Impact statement:**

The timing and rate of release of pharmaceuticals from advanced drug delivery systems is an important property that has received considerable attention in the scientific literature. Broadly, these mostly fall into two classes: controlled release with a prolonged release rate or triggered release where the drug is rapidly released in response to an environmental stimulus. This review aims to highlight the potential for developing adaptive release systems that more subtlety modulate the drug release profile through continuous communication with its environment facilitated through feedback control. By reviewing the key elements of this approach in one place (fundamental principles of nanomedicine, enzymatic nanoreactors for medical therapies and feedback-controlled chemical systems) and providing additional motivating case studies in the context of chronobiology, we hope to inspire innovative development of novel “chrononanomedicines.”

## Introduction

Innovative new technologies that increase the sophistication with which pharmaceuticals are released within the body are highly desirable to improve patient benefit from a treatment regimen.^1,2^ The potency of a chemotherapeutic in treating a medical condition generally requires the active drug to be bioavailable above a threshold concentration at the disease site for a long enough duration for it to be fully effective.^3^ This time period is usually much longer than the duration of action of a single dose. Poor patient adherence to a prescribed course of treatment is a common problem and can severely compromise its success.^4–6^ Therefore, novel drug formulations that can significantly prolong the effective duration time of a single dose will likely yield profound improvements in patient outcome.

**Figure 1.**
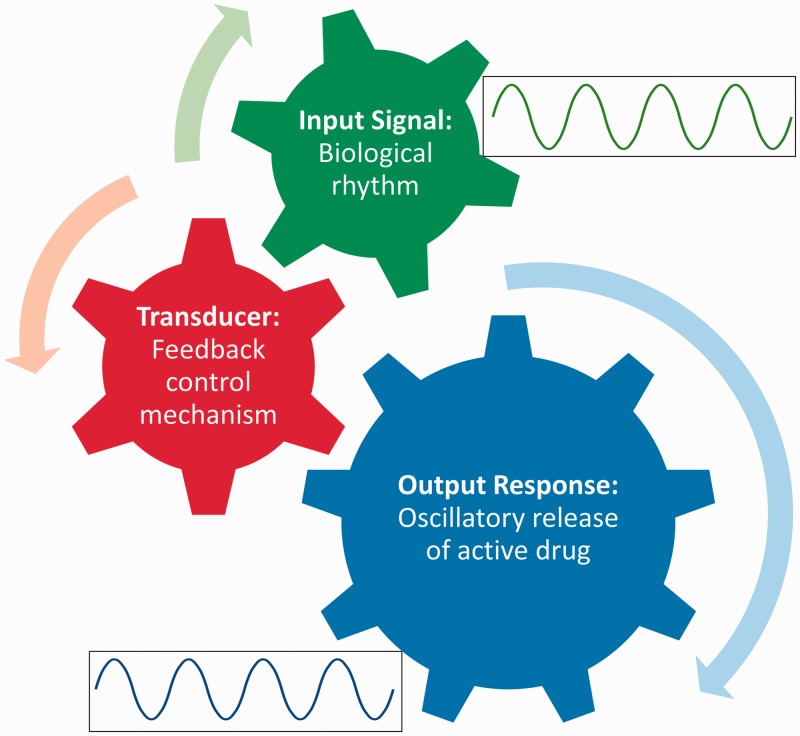
Design of feedback-responsive nanomedicines that adapt their release profiles to environmental cues such as the personalized biological rhythms of the patient. (A color version of this figure is available in the online journal.)

Nanomedicines have been investigated for controlled release formulations and have shown significant promise.^7–9^ However, most current such materials under development lack the sophistication to appropriately modulate their release in response to physicochemical cues from their local environment. This would require a mechanism of feedback control to be designed within the formulation. Feedback control would allow the delivery of the active drug to be adaptive and personalized to the specific disease environment, physiology, and pathology of individual patients. Furthermore, the physicochemical environment of each individual is not constant: our physiology changes in many oscillatory cycles across different, wide-ranging timescales. Many therapies would benefit from being “in tune” with these cycles to optimize their therapeutic effect (Figure 1).^10,11^

Feedback control in a biochemical system is fundamental to how life works.^12–16^ Understanding these features and harnessing them within a drug delivery system will therefore benefit from a bottom-up synthetic biology approach that encapsulates some metabolic (enzymatic) function.^17^ Here, we review progress towards this goal with an emphasis on integrating biological processes within a minimal system. We start by introducing basic principles of nanomedicines before specifically discussing therapeutic nanomedicines that integrate enzymatic function. We then introduce some basic concepts of chronobiology and the need for corresponding chronomedicines. This then leads into a review of feedback-controlled chemical systems that might be utilized in facilitating adaptive release nanomedicines, with a particular emphasis on those that are most likely to have appropriate biocompatibility. Finally, we summarize the challenge of successfully integrating these concepts into advanced drug delivery systems that might eventually deliver clinical benefit.

## Principles of nanomedicine

**Figure 2.**
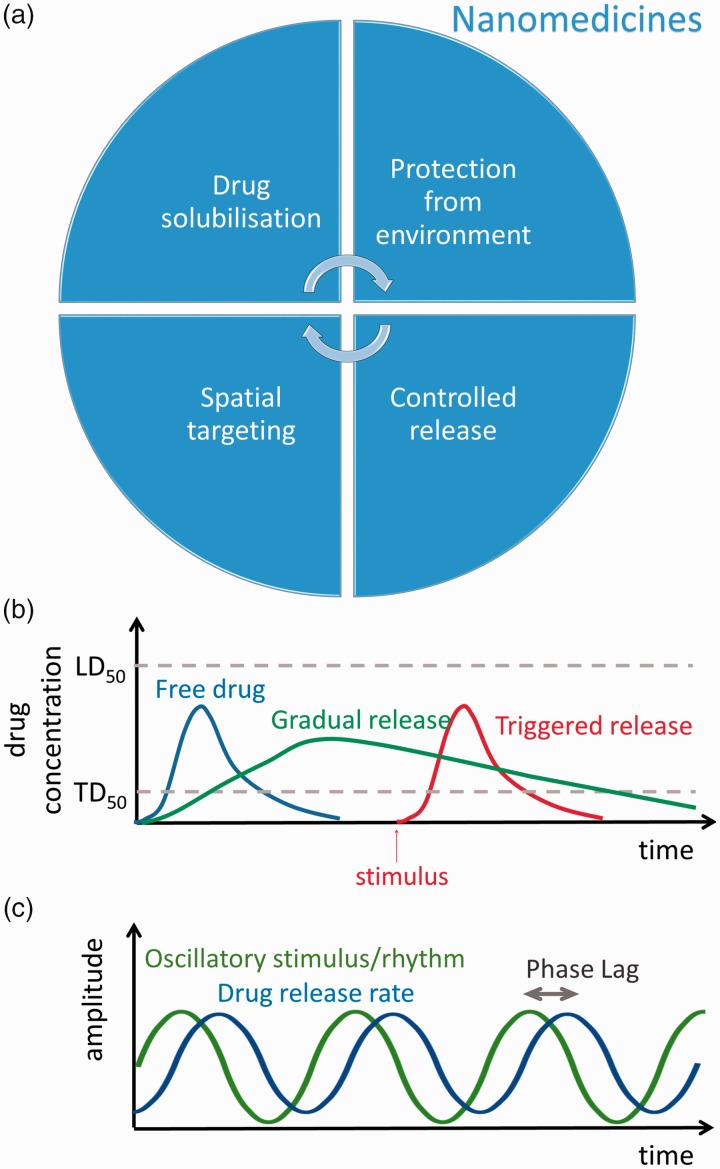
(a) Key functional components of engineered nanomedicines; (b) typical drug pharmacokinetics for free drug, delayed or triggered release systems and gradual release formulations (TD_50_ denotes the therapeutic or efficacious dose for 50% of patients; LD_50_ denotes the lethal or toxic dose for 50% of patients); (c) an example of a desirable drug release profile in response to a biological rhythm where the drug release rate exhibits “in tune” oscillations with a controllable phase shift. (A color version of this figure is available in the online journal.)

Formulation of active pharmaceutical ingredients (APIs) within nanoscale materials, at its core, aims to enhance pharmacokinetics and therapeutic index.^18^ Nanomaterial excipients can offer several potential advantages for the API (Figure 2(a)), including enhanced solubility and protection against metabolic degradation *en route* to its target. Similarly, by protecting the drug from early metabolism, this in turn protects healthy, non-target tissue from any toxic side-effects of the API. A classic example of this is the clinical nanomedicine DOXIL, which lowers the cardiotoxicity of the drug doxorubicin.^19^

Most of the advantages of nanomedicines can be attributed to enhanced temporal and spatial control of the API within the body.^20^ Conventional drug formulations are often delivered systemically to the whole body despite usually only being required in a particular organ or tissue, for example within a tumor for the case of cancer chemotherapeutics. The differential trafficking of nanoparticulates compared to small molecule entities within the body can be manipulated to yield enhanced concentrations of nanomedicines within target tissues, and hence increased concentrations of the API, and a concurrent decrease of API within off-target tissue.^21^ Nanomedicines are usually observed to accumulate within the liver and spleen, making nanomedicine therapies designed for these organs particularly promising, as well as enhanced delivery to sites of leaky vasculature and poor lymphatic drainage such as regions of inflammation or within solid tumors; this effect is referred to as enhanced permeability and retention.^22^ Nanomedicines can also modulate API biodistribution through an innate ability to cross biological barriers that may not be accessible to independent API molecules.^23^

Temporal control can be achieved through a variety of mechanisms that regulate the timing and kinetics of drug release from the nanoformulation (Figure 2(b)). Owing to the importance of maintaining free drug concentrations in the target tissue within the therapeutic range, a number of drug release systems, largely focused on achieving a degree of temporal control, have been investigated.

In its simplest form, temporal control can be obtained in terms of delayed or extended (sustained) release. Extended release formulations are usually achieved by loading the active drug into a material that acts as a depot from which the drug slowly liberates itself into the surrounding tissue.^24^ These materials can include macroscopic dermal patches but also nanoparticulate formulations that can be delivered intravenously to the circulatory system allowing transport and trafficking to the site of action.^25,26^ Such formulations aim to maintain a therapeutic concentration of drug at the disease site for as long a period as feasible from a single dose without risking patient toxicity (Figure 2(b)).

In instances of delayed release, the active component of a therapeutic compound is released at a time later than that of the initial administration and is often in response to a specific local environmental stimulus, i.e. a triggered release.^27^ A common example of delayed release is in oral delivery where the active drug requires protection from the low pH of the stomach but then must be released in the small intestine. This can be achieved by coating a therapeutic compound in a pH-responsive polymer where the polymer is insoluble in the low pH of the stomach, but when it passes to the higher pH environment of the small intestine, the polymer dissolves, releasing the drug.^28^ This gives rise to a pharmacokinetic profile similar to that of immediate release, but with a time delay (Figure 2(b)).

A range of other stimuli-responsive release mechanisms have been demonstrated, giving rise to a burst-release of encapsulated API in response to a specific environmental cue.^29^ These cues can be intrinsic triggers such as pH, redox potential or biomarker recognition, or extrinsic triggers such as from the application of magnetic or optical fields to the target tissue.

An additional possibility, which to our knowledge has not yet been explored, is to utilize intrinsic stimuli to produce a more adaptive release profile giving rise to more sophisticated temporal control within the delivery system (Figure 2(c)). Taking a biomimetic approach, this might be achieved through systems encapsulating metabolic function. Therefore, we consider progress in enzyme-encapsulating nanoreactor systems engineered for medical therapies.

## Nanoreactor and artificial cell systems for therapeutic delivery

Comparatively simplistic soft materials have provided initial success in bringing therapeutic nanoformulations into clinical trials and, in some cases, approved for clinical use. These include liposomes, polymer conjugates, and micelles. However, the simplicity of design in these systems precludes a high degree of sophistication of function. To achieve greater intricacy and complexity of function, we may look to nature for inspiration. To this end, enzymatic reactions in confined environments (e.g. within synthetic vesicles) are a promising route towards more elaborate, refined, and efficient temporal and spatial control.^30^ Efforts have been made to utilize principles of bottom-up synthetic biology to assemble artificial cell and organelle systems designed for advanced therapies.

### Artificial red blood cells

Within the field of oxygen therapeutics, both liposomes^31,32^ and polymersomes^33–36^ have been shown to successfully encapsulate hemoglobin. With the aim of creating an artificial red blood cell, hemoglobin-containing lipid vesicles have shown good efficacy in animal studies.^37^ Multi-compartmental vesicles, co-encapsulating hemoglobin with catalase or a reductant, e.g. homocysteine, further show an increase in oxygen uptake efficiency via the abolition of methemoglobin formation.^38^ However, these lipid-derived systems have associated shortcomings: low liposome stability, poor blood compatibility, reticuloendothelial system-induced degradation, and short blood circulation time. Therefore, polymersomes have been investigated as a means of protecting the encapsulants while working in a biological environment. Poly(ethylene oxide)-*block*-polybutadiene (PEO-*b*-PBD) and poly(ethylene oxide)-*block*-poly(ethyl ethylene) (PEO-*b*-PEE) polymersome nanoreactors are stable in blood plasma (temperature: 21°C) for up to five days, potentiating their use for successful oxygen replacement therapy.^34^ Reduced permeability, commonly associated with polymer membranes, significantly reduces oxygen binding (in comparison to free hemoglobin)^33^; however, incorporating OmpF as a transmembrane pore reduces this issue.^36^ Extensive research has been published on artificial red blood cell production, as an alternative to transfusion, leading to several reviews on this topic.^32,39–41^

### Reactive oxygen therapy

Superoxide dismutase (SOD) is an antioxidant enzyme known to catalytically degrade the reactive oxygen species (ROS) implicated in the pathogenesis of diseases such as rheumatoid arthritis, Alzheimer’s disease, and Parkinson’s disease. SOD has been successfully encapsulated in lipid^42^- and polymer-based^43,44^ vesicles. In rat models, PEGylation of liposomes was used to enhance blood circulation time, which ultimately resulted in greater SOD accumulation efficiency at targeted sites, and superior therapeutic effects, irrespective of dose or the type of enzyme.^45,46^ A hydrophobic variant of SOD, Ac-SOD, resulted in liposomes with SOD enzymes presented on their surface.^47^ Ac-SOD-liposomes showed a more efficient anti-inflammatory response in comparison to encapsulated enzyme due to their surface presentation. In the polymer system, alteration of PMOXA-*b*-PDMS-*b*-PMOXA block lengths was used to tune permeability, ultimately producing SOD-encapsulated nanoreactors with optimized properties.^48^ These antioxidant-based nanoreactor systems are expected for application in prevention of age-associated pathologies, including cataracts,^49^ cancer, age-associated macular degeneration, and Alzheimer’s disease.

Alternatively, nanoreactors that generate localized, high quantities of ROS present a strategy for photodynamic therapy.^50,51^ Polymersome nanoreactors containing a photosensitizer conjugated to bovine serum albumin, when excited by a specific wavelength of light, produce ROS, leading to cell death.^52^ Such nanoreactors are advantageous over previous photodynamic therapies due to the localized production of ROS without the release of the photosensitizer.^53,54^

**Figure 3.**
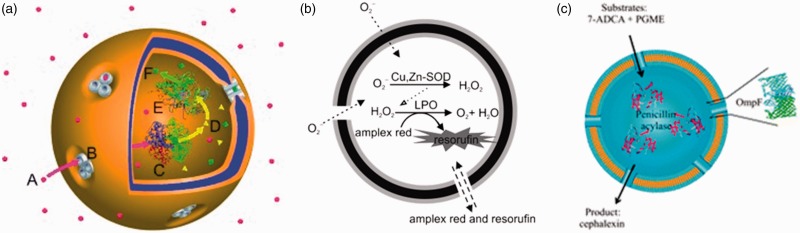
Enzyme-loaded therapeutic nanoreactors: (a) cascade reactions in a vesicle nanoreactor^44^; (b) ROS-theranostic nanoreactors contain SOD and LPO remove ROS and report oxidative stress through fluorescence^44^; (c) antibiotic nanoreactors for prodrug therapy convert inert prodrugs into the active therapeutic^55^—Reproduced by permission of The Royal Society of Chemistry. (a, b) Copyright © 2011 WILEY‐VCH Verlag GmbH & Co. KGaA, Weinheim: reproduced with permission of the publisher. (A color version of this figure is available in the online journal.)

In addition, theranostic nanoreactors have been devised, i.e. a product with both therapeutic and diagnostic qualities. Co-encapsulation of two distinct enzymes (SOD and lactoperoxidase) within a polymeric nanoreactor stimulated an enzymatic cascade, transforming ROS to H_2_O and O_2_, which, in turn, leads to production of a fluorescent reporter by conversion of amplex red to resorufin (Figure 3(a) and (b)). This ultimately led to a nanoreactor system capable of removing ROS while simultaneously monitoring ROS activity through fluorescence.^44^

### Enzyme replacement therapy

Nitric oxide (NO) is produced in the arterial endothelial layer and promotes artery wall relaxation, controlling blood flow and regulating blood pressure. Therefore, being NO deficient is a well-understood cause of arteriosclerosis, hyperglycemia, impotence and hypertension, among other diseases.^56^ The enzyme responsible for NO production is nitric oxide synthase (NOS), which has been successfully encapsulated in liposomes. These NOS-encapsulated nanoreactors were stable for a minimum of 15 days and maintained 75% enzyme activity, in comparison to free enzyme where the activity rapidly diminished.^56^

Nanoreactors for enzyme replacement therapy have also been developed for treatment of mitochondrial neurogastrointestinal encephalomyopathy. This is a genetic disease leading to an elevation of thymidine and deoxyuridine in blood plasma due to thymidine phosphorylase deficiency, causing toxicity primarily to the nervous and digestive systems.^57^ Successful encapsulation of thymidine phosphorylase has been demonstrated in polymersomes, where enzyme-catalyzed conversion of thymidine to thymine and deoxyribose 1-phosphate can take place. These nanoreactors were found to be both stable (blood serum, 37°C) and non-toxic (hepatocytes and macrophages).^58,59^

### Prodrug activation

Nanoreactors have been shown to hold promise in enhancing existing cancer therapeutics through directed enzyme prodrug therapy (DEPT). Artificial introduction of enzymes into the body is used as a means of converting a prodrug into the active therapeutic. However, the use of free enzyme leads to a high likelihood of invoking an immune response, leading to rapid clearance or potentially harmful adverse effects. To circumvent this problem, gene-DEPT (GDEPT) has been investigated, where an enzyme-encoding gene is delivered to cells to express the enzyme locally. Although this strategy significantly reduces systemic toxicity because toxin production is confined to the tissue to which the gene is delivered,^60^ an alternative and potentially more efficient strategy would be to encapsulate prodrug-activating enzymes within a nanoreactor. This can then be targeted to specific cells or tissues, ultimately eliciting their therapeutic effect upon docking. The advantages of enzyme encapsulation are that the circulation time following intravenous administration is increased and the likelihood of an immune response is greatly reduced. An example of this approach is the encapsulation of nucleoside hydrolase. Both lipid^61,62^ and polymer^63^ systems of this type have been reported: these nanoreactors convert non-toxic prodrugs 6-methyl-purine riboside and 2-fluoroadenosine to toxically active compounds 6-methylpurine and 2-fluroadenine, respectively. In each case, incorporation of OmpF channel proteins into the nanoreactor membranes facilitated enhanced permeability.

Proof of principle antibiotic prodrug-activating nanoreactors has also demonstrated promising efficacy. Penicillin acylase-encapsulating polymeric nanoreactors successfully converted prodrugs into an active drug *in situ* within bacterial cell culture (Figure 3(c)). Since the substrates have no antibiotic effect, the risk of adverse reactions is reduced and the localized efficacy of antibiotic effect will be increased. However, temporal control of antibiotic release was only partially achieved through the manipulation of fundamental parameters such as substrate concentration, vesicle size, and number of transmembrane OmpF channels present.^55^

### Cell-level therapies

An ambitious challenge for therapeutic nanoreactor technology is rooted in the repair of cell dysfunction through the mimicry of cell organelles or gene upregulation.^64–67^ Mimicking a natural organelle would not only have a resounding impact on the field of artificial cell (protocell) design, but it would also aid to revolutionize medical practice with regard to correcting cell-level disorders such as mitochondrial disease. At this moment in time, there has been no breakthrough in artificial organelle technology where the requirements to achieve such a feat is complicated on many fronts. The nanoreactor needs to be of an appropriate size to be taken into the cell; the *in situ* activity of encapsulated constituents needs to be preserved and the nanoreactor must be completely stable within the cell without being quickly broken down and recycled. If achieved, this technology would represent a significant milestone in *in vivo* cell organelle replacement and provide new modes of therapy.^68^

## Chronobiology and chronotherapeutics

Chronobiology is the study of the large number of biological processes that are not constant in time, but, instead, conform to a predictable rhythm defined by their frequency, phase and amplitude, and controlled by endogenous biological clocks.^11,69^ The time taken to complete these cycles can vary dramatically, from the subsecond pulsatile secretions of the neuroendocrine system, to the circadian production of melatonin in sleep–wake cycles, and beyond to longer circamensual cycles associated with ovulation (Table 1). The documentation and understanding of such biological rhythms have led to a growing importance being placed on the timing of drug dosing and the treatment of certain metabolic conditions according to circadian-based clocks.^11,70^

**Table 1:** . Range of biological rhythms and their associated times.^11^

Time category	Rhythm	Example
Short(*t* < 30 min)	Pulsatile (0.1 s < *t* < 1 s)Pulsatile (*t* ∼ 1 min)	Neural, cardiacCalcium, biochemical, insulin pulses
Intermediate(30 min < *t* < 6 days)	Ultradian (30 min < *t* < 20 h)Circadian (20 h < *t* < 28 h)Infradian (28 h < *t* < 6 days)	Mitotic, hormonal, insulin releaseSleep–wake, temperature,Cell cycle (cancer cells)
Long(*t* > 6 days)	Circaseptan (*t* ∼ 7 days)Infradian (*t* ∼ 30 days)Circannual (*t* ∼ 1 year)	ChronoimmunologyOvarianSeasonal

**Figure 4.**
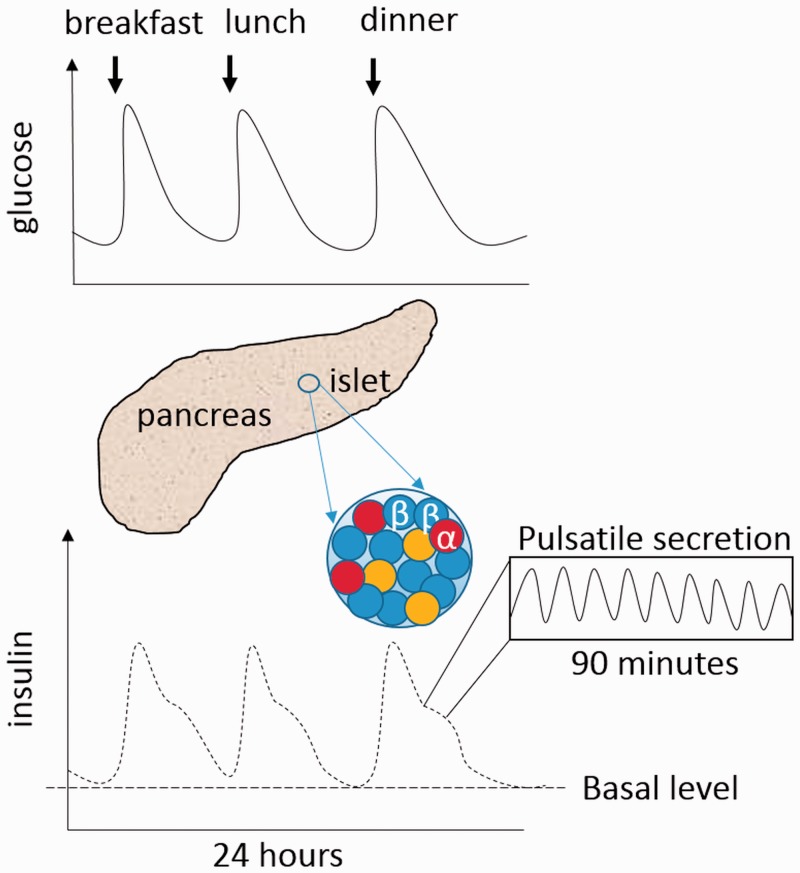
Example of a “closed loop” response to a physiological variable, i.e. insulin release from the β-cells in the pancreas in response to increased blood glucose levels. Inset shows pulsatile pattern of secretion. (A color version of this figure is available in the online journal.)

The need for advanced chronotherapeutics is not only necessitated by the strong association between certain diseases, e.g. asthma, angina, and cancer, and biological rhythms,^71–73^ but a greater therapeutic efficacy is also achieved when therapeutic compounds aim to mimic the pulsatile release of endogenous peptides, e.g. insulin, gonadotropin-releasing hormone (GnRH), and somatostatin.^74^ Insulin is a particularly interesting example. This hormone is secreted from the pancreas in response to the rise in glucose associated with having a meal.^75^ It then facilitates transport of glucose to the appropriate tissues and organs for utilization, before a basal level is restored once blood glucose levels fall (Figure 4). Type 1 diabetes is an autoimmune disease, where insulin-producing pancreatic β-cells are destroyed.^76^ In this instance, blood glucose increases to abnormally high levels. Treatment involves injections of insulin with meals; however, target glucose levels are difficult to maintain and hyper- or hypoglycemia can occur. As a result, extensive effort has focused on the development of a “closed-loop” drug delivery system that mimics the biological processes of the pancreas in response to blood glucose levels.^77,78^

Although the release of insulin is responsive, small amplitude discrete pulses of insulin are secreted from a healthy pancreas at an estimated rate of 5–15 min.^79,80^ One possible advantage for oscillatory secretion is that discontinuous exposure prevents down-regulation of insulin receptors and allows for receptor recovery. Type 2 diabetes is associated with resistance to insulin, and in this case, the rhythmic pattern is altered.^81^ Therapeutics that mimic pulsatile release not only reduce the amount of insulin delivered (in comparison to a continuous release system) but may enhance uptake by tissues.

GnRH is another hormone of clinical significance; it is produced and secreted by the hypothalamus in a pulsatile fashion. This peptide stimulates the release of other hormones, luteinizing hormone (LH) and follicle-stimulating hormone (FSH), from the anterior pituitary gland, which, in turn, circulate throughout the body stimulating the release of reproductive cells and hormones in the gonads.^82^ Although both men and women produce LH and FSH, biological feedback of these hormones in women controls the rate of GnRH release, which, in turn, mediates fertility to a monthly cycle.^83^ In cases of GnRH deficiency, common therapeutic procedure involves the intravenous administration of GnRH in a pulsatile fashion.^84^ Although successful in chronic cases of GnRH deficiency, the associated disadvantages of continuous intravenous administration, i.e. infection and inconvenience, suggests the need for fully implantable, autonomous alternatives.

Another example of therapeutics that would benefit from periodic delivery are agents which result in tolerance and/or reduced activity following continuous exposure. A clinical example of this is nitroglycerin, otherwise known as glyceryl trinitrate (GTN). GTN is rapidly absorbed in the body liberating NO, a vasodilator, and has long been used for the treatment and prevention of angina.^85^ However, the development of nitrate tolerance during sustained therapy is a major problem.^86^ In light of this, the current administration protocol consists of GTN application followed by a drug-free period to re-establish GTN sensitivity during each 24-h dosing period. The mechanisms associated with nitrate tolerance are still not completely understood, but a rhythmic drug delivery mechanism may prevent its onset.

The well-known circadian rhythms also play an important role in the periodicity of hormone release and activities including the sleep/wake cycle.^87^ These quasi 24-h oscillations regulate the behavior of diverse organisms, including bacteria, plants, and animals. The mammalian circadian clock is governed by a network of neurons located in the suprachiasmatic nucleus in the hypothalamus and is synchronized to the sun’s daily cycle. Disruption of this clock, for example, in sleep disorders, results in health problems that require treatment.^88,89^ It has also been well-documented that pharmacokinetic and pharmacodynamic factors associated with drug dosing and therapeutic outcome show variation depending on the time of day.^74,90^ Drug delivery, especially in the treatment of asthma, cardiovascular problems, and cancer, is evolving to take biological rhythms more heavily into account.^91,92^

Efforts to mimic natural rhythms in drug delivery has led to investigation of both “active techniques,” where an input of external energy, e.g. electrical,^93^ magnetism,^94^ ultrasound,^95^ or pressure,^96^ triggers drug release with the desired timing, and “passive techniques,” where drug release is temporally controlled without the need of an external stimuli through programmed disruption, layering, or utilization of feedback-driven processes within the drug delivery system.^97^

## Chemical feedback systems and their current applications

Feedback occurs when a process or system is regulated by its output and is used in biological systems to control the response to a stimulus, e.g. blood clotting, long-term potentiation (memory formation), or homeostasis.^98^ Feedback also facilities the internal switches and oscillators that provide a programmable temporal variation in biological properties.^12,99^ However, it is the fact that the interplay between feedback-regulated chemical reactions and physical processes can generate a specific function within a chemical system, such as biosensing and drug delivery, that has brought the area so much attention from chemists in recent years.^100–103^

It is well understood that chemical oscillators can be generated by combining positive and negative feedback.^104^ Positive feedback amplifies a stimulus and can arise when a reaction is catalyzed by its products (autocatalysis). Negative feedback suppresses the autocatalytic process, and for oscillations to arise, the negative feedback must be delayed relative to the positive feedback. The most well-characterized chemical oscillator based on these principles is the Belousov-Zhabotinskii (BZ) reaction, where, in the presence a metal-ion catalyst, an acidified bromate solution oxidizes an organic substrate (typically malonic acid) in a series of reactions.^105^ The BZ reaction was utilized in the creation of the first self-oscillating polymer gels, where periodic redox oscillations cause rhythmic volume changes in a catalyst-loaded gel when immersed in a substrate-rich, acidic solution.^106,107^ The BZ oscillatory reaction has also been successfully encapsulated and confined within emulsion droplets, which exhibit a rich range of phenomena including physical morphogenesis.^108,109^ However, the BZ reaction involves harsh oxidation chemistry rendering it incompatible with biological applications.

Of greater interest for potential applications are the pH oscillators.^110^ These may be used to change the protonation state of a drug, and therefore its permeability across a membrane, or drive periodic volume changes in a pH-responsive gel, thereby liberating a drug.^111^ In one model system, the pH-oscillating medium containing benzoic acid was placed next to a lipophilic ethylene vinyl acetate copolymer membrane.^112^ It was assumed that the oscillations in pH (pH 2–7) would result in the intermittent diffusion of benzoic acid (uncharged) across the lipophilic membrane. However, the inclusion of benzoic acid damped the oscillations, and a steady state pH close to the pKa of the drug was obtained. These findings demonstrate that pH oscillators are limited in their use in rhythmic drug delivery systems unless they can be designed to overcome drug buffering effects.^113^

**Figure 5.**
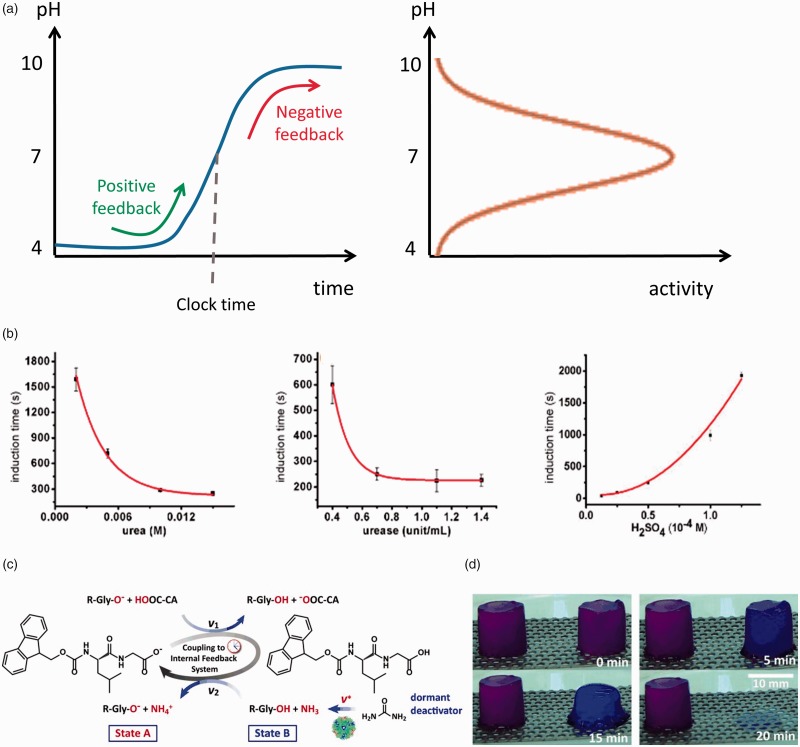
Urea–urease autocatalytic feedback. (a) The emergence of a clock time in pH-switching triggered by the urea-urease reaction and its relation to pH-dependent enzyme activity; (b) variation of clock time (induction time) with urea, urease, and sulfuric acid concentration starting conditions^117^; (c) pH-dependent hydrogels with programmable lifetimes (d) controlled by the clock time of a urea–urease reaction.^118^ (c, d) © 2015 WILEY‐VCH Verlag GmbH & Co. KGaA, Weinheim, reproduced with permission of the publisher. (A color version of this figure is available in the online journal.)

Enzyme-catalyzed reactions provide the most viable option for biocompatible feedback. However, only a few robust and reproducible enzyme-catalyzed reactions have so far been shown to exhibit feedback *in vitro*.^114–116^ One well-established example is the urease-catalyzed hydrolysis of urea to ammonia and carbon dioxide.^117^ Urease has been discovered in a variety of difference sources, including bacteria, fungi, and plants. The enzyme activity depends on pH, as variations in pH alter the protein-binding site conformation, and the reaction has a maximum rate at pH 7 (Figure 5). If the starting pH of the reaction is lowered via the addition of acid, then, after a lag time determined by the initial conditions, the reaction rate accelerates (positive feedback). Once the pH passes its activity maximum, the reaction rate will begin to slow and stabilize at a new pH <10 (negative feedback). When conducted in a closed reactor, the urease reaction remains at an acidic pH (∼pH 4) for a particular amount of time before rapidly changing to a basic pH (∼pH 10). Chemical systems that display a sharp change in state after a programmable period of time are known as clock reactions (in contrast to the periodic biological clock). The clock time and final pH of the urea–urease reaction can be finely controlled through the initial concentrations of acid, urea, and urease (Figure 5(b)).^119^ Besides periodic chemical oscillators, clock reactions are of interest in the design of adaptive nanomedicines in their own right: their tuneable, environment-responsive clock times allow an intrinsic programmable time delay that could, for example, allow a secondary drug dose to be delivered following metabolism of a primary dose, or a delayed release to deliver at a specific timing within a biological cycle.

There is increasing interest in the exploitation of enzymatic feedback coupled with smart materials, i.e. hydrogels, polymers, or polymersomes, that degrade, swell, or increase permeability, in response, for example, to a change in pH.^120,121^ The switch to high pH in the urea–urease reaction has been coupled to a pH-sensitive hydrogel (Figure 5(c)), resulting in the degradation of the gel after a programmable time lag.^118^ Oscillations were obtained through autocatalytic generation of the enzyme trypsin and the reaction was used to drive periodic disassembly of coacervates.^122^ The glucose–glucose oxidase reaction has been subject to great attention in recent years, owing to its potential as the autonomous “brain” of responsive insulin release.^123^ In this reaction, glucose oxidase drives catalytic conversion of glucose into hydrogen peroxide and D-glucono-δ-lactone, which subsequently hydrolyses into gluconic acid, thereby lowering the pH. This acid production was combined with gel-volume hysteresis to obtain periodic changes in pH that drove delivery of insulin across a membrane.^124^

Although enzyme reactions have been widely exploited in healthcare applications, feedback-driven responses have been demonstrated to a lesser extent and may require significant tuning to achieve the desired response. Currently, all known homogeneous pH oscillator systems necessitate the need for open flow conditions, because typically at least one of the substrates is fully consumed in the initial pH switch.^125^ In the urea–urease reaction, however, only partial consumption of urea occurs, highlighting its potential as the first batch pH oscillator upon addition of the necessary delayed negative feedback.^117^ Although this reaction has been coupled with the acid-autocatalytic glucose oxidase reaction in a number of applications, to date oscillations have not been reported.^126^ In theory, the cooperation of such reactions, coupled to a secondary response, e.g. drug release/activation with a responsive and/or tuneable release mechanism, could hold the key to a fully autonomous and oscillatory drug delivery system.

## The challenge and future outlook

With initial drug nanoformulations successfully translated to the clinic, early proof-of-concept examples of enzyme-loaded vesicles and a developing understanding of the systems chemistry of feedback-responsive chemical reactions, the groundwork is in place for the design of novel adaptive delivery systems limited primarily by our imaginations and creativity. For example, feedback in enzymatic reactions (e.g. urea/urease) can modulate the pH of their environment, which could be linked to changes in the material properties of the drug carrier. A wide range of pH-responsive materials have been developed in the literature and proof of concept for urea/urease temporally controlled hydrogels is an exemplar of such a system.

Challenges will undoubtedly be encountered in the realization of such functionally complex systems. Protocols will need to be developed that facilitate optimal co-encapsulation, providing non-denaturing conditions for the enzyme in the presence of high drug loading. Potential chemical cross-talk between the enzymes and drugs will also need to be understood and controlled unless more complex nanomedicine architectures such as multicompartment vesicles are employed.^127–130^ Fundamental studies of feedback response in confined and compartmentalized materials, an endeavor that is also of significance for the development of artificial cells in synthetic biology, will provide further underpinning conceptual understanding of how to optimally engineer adaptive-release nanomedicines.

While traditional approaches to personalized medicines have viewed this challenge through a pretreatment refinement of the therapeutic regimen by the clinician, informed by information about the specific pathological or physiological characteristics of the patient,^131^ adaptive nanomedicines hold long-term promise for intrinsic personalization through communication with and modulation by the specific pathophysiology encountered, including adaption and synchronization to relevant chronobiological cycles, where beneficial. Materials scientists will need to collaborate closely with their biomedical and pharmaceutical counterparts to identify promising combinations of environmental signal, feedback-responsive metabolic process and API to be delivered for specific disease therapies. The scene is set for inception of the next generation of innovative nanomedicines with temporal-control over release that more closely imitates the environmental communication and dynamically changing response of biological systems.
